# Influenza epidemics observed in primary care from 1984 to 2017 in France: A decrease in epidemic size over time

**DOI:** 10.1111/irv.12620

**Published:** 2019-01-18

**Authors:** Cécile Souty, Philippe Amoros, Alessandra Falchi, Lisandru Capai, Isabelle Bonmarin, Sylvie van der Werf, Shirley Masse, Clément Turbelin, Louise Rossignol, Ana‐Maria Vilcu, Daniel Lévy‐Bruhl, Bruno Lina, Laëtitia Minodier, Yves Dorléans, Caroline Guerrisi, Thomas Hanslik, Thierry Blanchon

**Affiliations:** ^1^ Sorbonne Université INSERM Institut Pierre Louis d’Épidémiologie et de Santé Publique (IPLESP) Paris France; ^2^ EA7310, Laboratoire de Virologie Université de Corse‐Inserm Corte France; ^3^ Department of Infectious Diseases Santé publique France Saint‐Maurice France; ^4^ Institut Pasteur Unité de Génétique Moléculaire des Virus à ARN Paris France; ^5^ Institut Pasteur Centre Coordonnateur du Centre National de Référence des virus des infections respiratoires (dont la grippe) Paris France; ^6^ UMR CNRS 3569 Paris France; ^7^ Université Paris Diderot Sorbonne Paris Cité Unité de Génétique Moléculaire des Virus à ARN Paris France; ^8^ Laboratoire de Virologie Hospices Civils de Lyon Institut des Agents Infectieux (IAI) Centre National de Référence des virus respiratoires (dont la grippe) Centre de Biologie et de Pathologie Nord Groupement Hospitalier Nord Lyon France; ^9^ Université de Lyon Virpath, CIRI, INSERM U1111 CNRS UMR5308 ENS Lyon, Université Claude Bernard Lyon 1 Lyon France; ^10^ Université de Versailles Saint‐Quentin‐en‐Yvelines UVSQ UFR de Médecine Versailles France; ^11^ Service de Médecine Interne Hôpital Ambroise Paré Assistance Publique – Hôpitaux de Paris APHP Boulogne Billancourt France

**Keywords:** epidemics, influenza, influenza‐like illness, primary care, surveillance

## Abstract

**Background:**

Epidemiological analysis of past influenza epidemics remains essential to understand the evolution of the disease and optimize control and prevention strategies. Here, we aimed to use data collected by a primary care surveillance system over the last three decades to study trends in influenza epidemics and describe epidemic profiles according to circulating influenza viruses.

**Methods:**

Influenza‐like illness (ILI) weekly incidences were estimated using cases reported by general practitioners participating in the French *Sentinelles* network, between 1984 and 2017. Influenza epidemics were detected by applying a periodic regression to this time series. Epidemic (co‐)dominant influenza virus (sub)types were determined using French virology data.

**Results:**

During the study period, 297 607 ILI cases were reported allowing the detection of 33 influenza epidemics. On average, seasonal epidemics lasted 9 weeks and affected 4.1% of the population (95% CI 3.5; 4.7). Mean age of cases was 29 years. Epidemic size decreased over time by ‐66 cases per 100 000 population per season on average (95% CI −132; −0.2, *P* value = 0.049) and epidemic height decreased by ‐15 cases per 100 000 (95% CI −28; −2, *P* value = 0.022). Epidemic duration appeared stable over time. Epidemics were mostly dominated by A(H3N2) (n = 17, 52%), associated with larger epidemic size, higher epidemic peak and older age of cases.

**Conclusions:**

The declining trend in influenza epidemic size and height over the last 33 years might be related to several factors like increased vaccine coverage, hygiene improvements or changing in influenza viruses. However, further researches are needed to assess the impact of potential contributing factors to adapt influenza plans.

## 1 INTRODUCTION

Influenza is a common respiratory infectious disease, manifested typically by influenza‐like illness (ILI) usually defined by sudden onset of fever, myalgia and respiratory signs.^1^ Although morbidity and mortality attributed to influenza epidemics vary depending on circulating strains,^2^ influenza epidemics cause each year between 3 and 5 million of severe cases and between 250 and 500 thousands of deaths around the world.^3^

In France, the yearly impact of winter acute respiratory illness was estimated between 22 and 29% of the population over the 2012‐2017 period.^4^, ^5^ The excess respiratory deaths due to influenza seasonal epidemics were estimated to 2.9 per 100 000 population each year.^6^ The socio‐economic impact was estimated at 2.9 (±2.5) days of work lost per person and per flu episode,^7^ for a total cost of $2.6 billion, of which $2.3 billion indirect costs (mainly due to loss of productivity) and $0.3 billion direct costs spent for medical care.^8^

The health and socio‐economic impact of influenza epidemics, along with the need to assess the impact of influenza vaccine in the field, justify its surveillance. Real‐time monitoring of influenza epidemics is used by authorities and healthcare professionals to implement or adjust interventions. However, retrospective analysis of influenza epidemics over a long period of time seems essential to better understand disease seasonality, variation of long‐term trend and potentially forecast future trends. This allows providing information to adapt overall strategies for prevention and control and to plan management of healthcare facilities.

Influenza surveillance in France is coordinated by French national public health agency (ie, *Santé publique France*) including hospitals, laboratories and primary care networks.^9^ The French primary care surveillance network—called *Sentinelles*, participate in monitoring influenza epidemics since 1984 through a sample of general practitioners (GPs) reporting on a weekly basis all ILI cases seen in consultation.^10^ ILI definition used had a high predictive value for influenza although other respiratory viruses could cause ILI.^11^ This definition has remained unchanged over the period, allowing study of trends in ILI. There were no steady influenza virological database available in primary care since 1984, as this laboratory surveillance has evolved, first coordinated by the GROG—*Groupes Régionaux d'Observation de la Grippe*, before 2014^12^ and then by the *Sentinelles* network.^13^, ^14^

The work presented here aims to study evolution of the dynamics of influenza epidemics observed in primary care in France between winters 1984/85 and 2016/17 and to describe epidemic profiles according to circulating influenza viruses.

## 2 METHODS

### 2.1 Study population: influenza‐like illness cases

The French *Sentinelles* network (http://www.sentiweb.fr) is a real‐time epidemiologic surveillance system in primary care created in 1984.^10^ A sample of French GPs located all over the country participates on a voluntary basis to data reporting. In 2017, 458 sentinel general practitioners (SGPs) out of the 61 535 French GPs (0.8%) participated in the surveillance varying from 0.36% (228/62 775) in 2004 to 1.0% (509/49 314) in 1988. A previous study has analysed differences between SGPs and all French GPs.^15^ SGPs were similar to other GPs for age and practice of a complementary medicine, but they differed in a number of ways: they were more frequently males, their number of consultation by week were slightly higher and were not equally spread over the territory.

Since late 1984, SGPs reported throughout the year weekly numbers of ILI cases seen in consultation and described these cases.^16^ An ILI case is defined as a sudden onset of fever over 39°C with myalgia and respiratory symptoms. The description form for ILI cases has changed over years adding specific questions linked to public health issues. They concern: age, sex, influenza seasonal vaccination status (since September 1986) and whether the delay between vaccination and onset of symptoms is more than 3 weeks (2 weeks since September 2014), hospitalization request (since September 1997), risk factors for influenza complications except age (since September 2009), antiviral prescription (since September 2009), antibiotic prescription and its name (since September 2013).

The study was performed using data collected from 1984 week 40 to 2017 week 25.

The protocol was conducted in agreement with the Helsinki declaration. We obtained authorization from the French Data Protection Agency (CNIL, registration number #471393).

### 2.2 Incidence estimation

In France, the size of the population covered by GPs is unknown because there is no mandatory practice register.^15^ Thus, the total number of ILI cases by week (weekly incidence) is estimated by multiplying the mean number of reported cases per participating SGP for a given week by the total number of practicing GPs. To take into account the regional disparities of SGPs density, weekly incidence was first estimated at the regional level (Nomenclature of territorial units for statistics—NUTS 2 level 17), and then summed to obtain the national estimates. Incidence rates (per 100 000 population) were obtained by dividing incidences by yearly population size (census data). Age‐specific incidence rates were estimated for the following age groups: 0‐4 years old, 5‐14 years, 15‐64 years, 65 years and older. Confidence intervals (CI) were estimated assuming that cases reported by SGPs follow a Poisson distribution.^18^

### 2.3 Determination of epidemic period

Influenza epidemic detection method was based on a regression model which fits non‐epidemic data to predict a non‐epidemic baseline. ILI non‐epidemic baseline were estimated by applying a periodic regression model including a linear trend, annual and semi‐annual periodic terms on weekly ILI incidence rates below a cut‐off value (defined at 279 cases per 100 000 population).^19^ Epidemic thresholds were defined as the estimated baseline's upper 90% prediction bound. The epidemic is declared when at least two consecutive weekly incidence rates exceed the threshold. This epidemic detection method was selected based on its performance, evaluated in a recent study.^20^

Each epidemic was named by its influenza season (ie, 2011/12 epidemic, refers to the epidemic period having occurred between September 2011 and August 2012).

### 2.4 Description of epidemic profiles

In the analyses, the 2009 pandemic has been considered aside from the seasonal epidemics.

The epidemic peak is defined as the highest weekly ILI incidence during the epidemic period. Epidemic size refers to weekly cumulated incidence rates during the epidemic period. Change in epidemic size and height over time was studied using linear regression where cumulated ILI incidence rates by epidemic were predicted by a time variable (eg, year).

Epidemics were classified into three groups according to the time of the influenza season started—called “start period.” Breaks were defined using the first and the third quartiles of the start of the 32 seasonal epidemics. Associations between start period and epidemic size, height and duration were studied using variance analysis.

For each epidemic, the age‐specific burden of illness was assessed with the relative illness ratio (RIR).^21^ This ratio is defined as the contribution of the age group *i* to ILI cases divide by its contribution to the general population:

$$\text{RIR}=\frac{C_{i}/\sum_{i}C_{i}}{N_{i}/\sum_{i}N_{i}}$$

where *C*_*i*_ is the number of ILI cases in age group *i* and *N*_*i*_ the total population of age group *i*.

This ratio allows assessing the under‐ or over‐representation of an age group among ILI cases: a ratio above one indicates an excess risk. Besides, being standardized on epidemic size, it can be compared across epidemics. Confidence intervals (CI) were estimated with the exact Poisson method.^22^

### 2.5 Influenza viruses circulating

In France, there was no shared and steady database about circulating influenza viruses in primary care. To obtain quantitative data on influenza viruses circulating in primary care during past epidemics in France, we consulted several sources: the French national public health agency (data published in the *Bulletin Epidemiologique Hebdomadaire,* http://invs.santepubliquefrance.fr/beh/index.html ), a published article,^12^ and for two seasons, as no data were published by the French authorities, we used French virology data reported by the Centers for Disease Control and Prevention in the Morbidity and Mortality Weekly Report (https://www.cdc.gov/mmwr/index.html). Virological data sources for each season are detailed in Table 1.

**Table 1:** Characteristics of influenza epidemics in primary care detected by the *Sentinelles* network, 1984/85 to 2016/17, France

Epidemic	Starting week	Ending week	Duration (weeks)	Peak week	Cumulated incidence [95% CI] (in million cases)	Cumulated incidence rates [95% CI] (cases per 100 000 population)	Peak incidence rate (cases per 100 000 population)	Dominant or co‐dominant virus(es)	B lineage
1984/85	1985w03	1985w14	12	1985w05	4.3 [3.9; 4.7]	7758 [7072; 8444]	1155	A(H3N2)^a^	–
1985/86	1986w02	1986w12	11	1986w07	3.4 [3.1; 3.7]	6160 [5663; 6657]	886	A(H3N2)^a^^,^ b	–
1986/87	1987w04	1987w09	6	1987w06	1.3 [1.2; 1.4]	2415 [2244; 2586]	533	A(H1N1)^a^	–
1987/88	1988w09	1988w15	7	1988w11	1.4 [1.3; 1.4]	2432 [2280; 2584]	566	B^a^	Victoria
1988/89	1988w46	1989w02	9	1988w50	4.6 [4.4; 4.8]	8227 [7920; 8534]	1793	A(H1N1)^a^	–
1989/90	1989w48	1990w06	11	1989w51	4.6 [4.4; 4.9]	8207 [7765; 8649]	1463	A(H3N2)^a^	–
1990/91	1991w06	1991w10	5	1991w08	0.8 [0.7; 0.9]	1386 [1256; 1516]	381	B^a^	Yamagata
1991/92	1991w49	1992w06	10	1991w51	2 [1.8; 2.2]	3542 [3236; 3848]	666	A(H3N2)^b^	–
1992/93	1993w03	1993w13	11	1993w06	1.9 [1.7; 2]	3268 [3048; 3488]	500	B^b^	Victoria
1993/94	1993w46	1994w01	8	1993w49	3.1 [3; 3.2]	5402 [5176; 5628]	1565	A(H3N2)^a^^,^ b	–
1994/95	1995w11	1995w18	8	1995w14	1.1 [1; 1.2]	1925 [1782; 2068]	431	A(H3N2),B^a^	Yamagata
1995/96	1995w47	1996w02	8	1995w51	2.8 [2.7; 2.9]	4818 [4594; 5042]	1299	A(H1N1),A(H3N2)^a^	–
1996/97	1996w48	1997w05	10	1996w51	3 [2.8; 3.2]	5175 [4905; 5445]	1106	A(H3N2)^a^	–
1997/98	1998w06	1998w17	12	1998w14	2.4 [2.2; 2.6]	4178 [3848; 4508]	547	A(H3N2)^a^	–
1998/99	1998w53	1999w11	12	1999w07	3.3 [3; 3.5]	5581 [5173; 5989]	896	A(H3N2)^a^	–
1999/00	1999w49	2000w06	10	2000w01	3.3 [3.1; 3.5]	5593 [5204; 5982]	922	A(H3N2)^a^	–
2000/01	2000w50	2001w07	10	2001w05	1.6 [1.4; 1.7]	2629 [2310; 2948]	471	A(H1N1)^a^	–
2001/02	2002w01	2002w08	8	2002w04	2.3 [2.1; 2.5]	3893 [3509; 4277]	848	A(H3N2)^a^	–
2002/03	2003w05	2003w15	11	2003w07	1.5 [1.3; 1.7]	2533 [2151; 2915]	396	B^a^	Victoria
2003/04	2003w45	2004w01	9	2003w49	2.8 [2.5; 3.1]	4667 [4168; 5166]	928	A(H3N2)^c^	–
2004/05	2005w03	2005w12	10	2005w06	3.1 [2.9; 3.3]	5106 [4708; 5504]	939	A(H3N2)^a^^,^ c	–
2005/06	2006w04	2006w13	10	2006w06	1.6 [1.4; 1.8]	2598 [2312; 2884]	421	A(H1N1),B^a^^,^ c	Victoria
2006/07	2007w03	2007w09	7	2007w06	2.1 [1.9; 2.3]	3398 [3100; 3696]	815	A(H3N2)^a^^,^ c	–
2007/08	2008w02	2008w10	9	2008w06	2.1 [1.9; 2.3]	3468 [3168; 3768]	615	A(H1N1),B^a^^,^ c	Yamagata
2008/09	2008w51	2009w08	10	2009w04	2.8 [2.5; 3.1]	4459 [3993; 4925]	868	A(H3N2)^c^	–
2009/10	2009w37	2009w52	16	2009w49	3.5 [3.2; 3.8]	5515 [5043; 5987]	754	A(H1N1)pdm09^a^^,^ c	–
2010/11	2010w51	2011w07	9	2011w01	2.2 [2; 2.4]	3491 [3231; 3751]	490	A(H1N1)pdm09, B^a^^,^ c	Victoria
2011/12	2012w05	2012w12	8	2012w08	1.4 [1.3; 1.6]	2276 [2060; 2492]	452	A(H3N2)^a^^,^ c	–
2012/13	2012w51	2013w11	13	2013w05	3.5 [3.3; 3.8]	5531 [5124; 5938]	770	B^a^^,^ c	Yamagata
2013/14	2014w05	2014w09	5	2014w07	0.8 [0.7; 0.9]	1284 [1163; 1405]	325	A(H1N1)pdm09, A(H3N2)^a^	–
2014/15	2015w03	2015w11	9	2015w06	2.8 [2.7; 3]	4413 [4171; 4655]	827	A(H3N2)^a^	–
2015/16	2016w04	2016w14	11	2016w11	2.3 [2.1; 2.4]	3465 [3220; 3710]	467	B^a^	Victoria
2016/17	2016w50	2017w05	8	2017w03	1.8 [1.7; 1.9]	2720 [2535; 2905]	410	A(H3N2)^a^	–

Data source used for determination of virological dominance and co‐dominance by influenza epidemic in France:

a French national public health agency.

b Morbidity and Mortality Weekly Report (Centers for Disease Control and Prevention).

c Mosnier et al.^12^

Using these collected data, virological dominance and co‐dominance by influenza epidemic was established using the following decision rule adapted from literature.^23^, ^24^, ^25^ The main circulating virus type or subtype was considered as “dominant” if: (a) it accounted for 70% or more of all isolates during the season or (b) it accounted between 40% and <70% of all isolates and the second most common virus type or subtype accounted for <30%. Two types or subtypes are considered as “co‐dominant” if the main circulating virus accounted between 40% and <70% of all isolates and the second most common accounted for 30% or more of all isolates.

We distinguish A(H1N1) pre‐ and post‐pandemic subtypes (eg, circulating before and after the 2009 pandemic), denoted A(H1N1) and A(H1N1)pdm09, respectively.

## 3 RESULTS

### 3.1 Epidemic profiles

**Figure 1:**
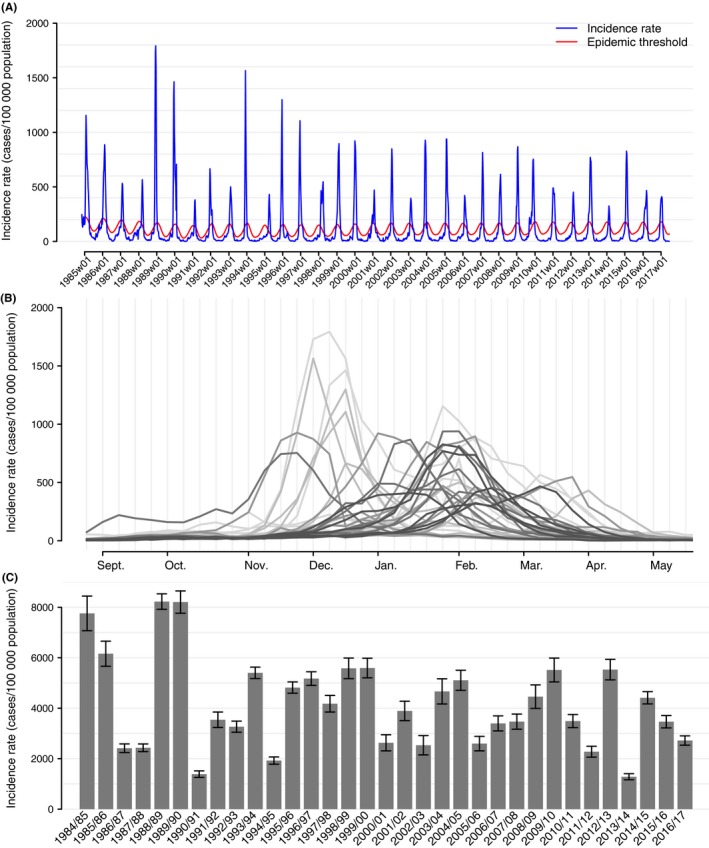
Incidence of influenza‐like illness estimated per 100 000 population between 1984 and 2017; (A) weekly continuous series; (B) layered epidemic (the clearer the line, the older the epidemic); (C) cumulative ILI incidence rate by epidemic and 95% confidence intervals, *Sentinelles* network, France

Data collected by the *Sentinelles* network allowed the detection of 32 influenza seasonal epidemics and one pandemic during the 33 monitored seasons (Figure 1 and Table 1).

When excluding the 2009 pandemic, mean epidemic duration was 9.3 weeks (95% CI 8.6; 10.0). Mean start of epidemics is the 1st of January, with inter‐quartile range between the 9th of December and the 20th of January (Figure 1B). The average cumulative epidemic incidence rate was estimated to 4125 cases per 100 000 population (95% CI 3492; 4758), corresponding to an average epidemic incidence of 2.4 million cases (95% CI 2.1; 2.8) (Figure 1C and Table 1). On average, the epidemic peak occurred 4 weeks after the epidemic start, with a mean weekly incidence rate of 773 cases per 100 000 population (Figure 1A).

We highlighted a positive correlation between epidemic size and duration (ρ = 0.54, *P* value < 0.002), epidemic size and peak height (ρ = 0.87, *P* value < 10^−9^). However, there was no evidence of association between epidemic duration and peak incidence (ρ = 0.17, *P* value = 0.36). Influenza epidemic start period and epidemic size were associated (variance analysis, *P* value < 3.10^−4^). Compared to “winter” epidemics (starting between the 9th of December and the 18th of January), on average “early” epidemics (starting before the 8th of December) were larger (+1418 cases per 100 000 population; 95% CI 153; 2682) and “late” epidemics (starting after the 21th of January) were smaller (−1833 cases per 100 000 population; 95% CI −3051; −615). Likewise, peak incidence rate was higher for “early” epidemics and lower for “late” epidemics compared to “winter” epidemics (*P* value = 2.10^−6^; +483 [265; 700] and −292 [−502; −82] cases per 100 000 population, respectively).

**Figure 2:**
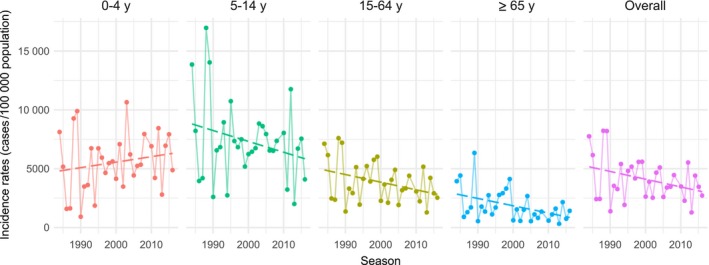
Incidence of influenza‐like illness estimated per 100 000 population by epidemic for four age groups and overall, with linear regression lines, from 1984/85 to 2016/17, *Sentinelles* network, France

Epidemic size declined over the 32 seasonal epidemics studied by 66 cases per 100 000 population per season on average (95% CI −132; −0.2, *P* value = 0.049), along with peak incidence rate with a coefficient of −15 cases per 100 000 population per season on average (IC 95% −28; −2, *P* value = 0.022). Among age groups, we highlighted a decrease in ILI incidence over time for adults (15‐64 years) and elderly (≥65 years) (respectively −65 cases per 100 000 population; 95% CI −127; −2, *P* value = 0.043 and −62; 95% CI −109; −14, *P* value = 0.012) (Figure 2). The slopes coefficients were not significantly different from zero in the other age groups (<5 years: 47 cases per 100 000 population, 95% CI −46; 140; 5‐14 years: −93 cases per 100 000 population, 95% CI −217; 32). We did not bring out changes in epidemic duration over the period (*P* value = 0.93).

### 3.2 Influenza viruses circulating

Influenza virus dominance and co‐dominance established for the 33 influenza epidemics were reported in Table 1. For 22 epidemics (67%), the dominant viruses were A type, including 17 (52%) epidemics dominated by A(H3N2), for six epidemics (18%), type B was dominant and for four epidemics (12%) viruses A and B were co‐dominant. Lastly, one pandemic influenza was observed in 2009/10, dominated by A(H1N1)pdm09.

**Figure 3:**
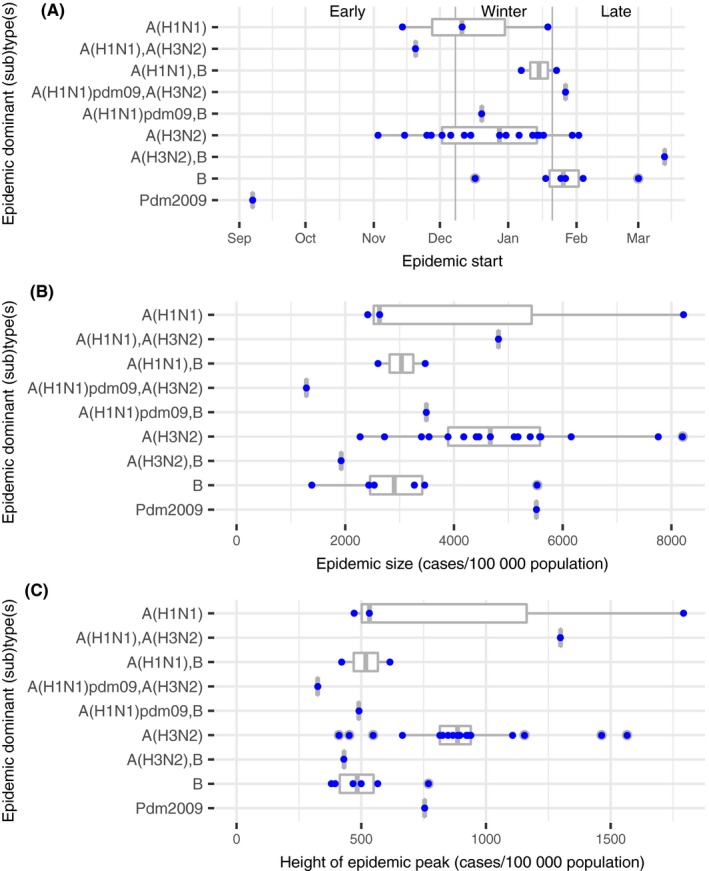
Characteristics of influenza epidemics grouped by viral dominant or co‐dominant (sub)types; (A) epidemic starting period; (B) cumulative incidence rate during epidemics; (C) incidence rate at epidemic peak; from 1984/85 to 2016/17, *Sentinelles* network, France

Epidemics dominated by A(H3N2) were associated with a larger size (4855 (95% CI 4044; 5665) vs 3298 (95% CI 2313; 4283) cases per 100 000 population, *P* value = 0.01) and higher peak (900 (95% CI 742;1057) vs 631 cases per 100 000 population (95% CI 410; 851), *P* value = 0.04) compared to all others epidemics. Conversely, epidemics dominated or co‐dominated by virus B were smaller (3010 (95% CI 2197; 3822) vs 4632 (95% CI 3801; 5462) cases per 100 000 population, *P* value = 0.005) and had a lower peak incidence rate (504 (95% CI 419; 598) vs 896 cases per 100 000 population (95% CI 725; 1067), *P* value = 2.10^−3^) (Figure 3).

None of the 10 epidemics dominated or co‐dominated by type B viruses started early in the season. Among the “late” epidemics, 6/9 (67%) were dominated or co‐dominated by type B. Moreover, among the “early” epidemics, 6/8 (75%) were dominated by A(H3N2) viruses (Figure 3).

### 3.3 Characteristics of ILI cases reported by SGPs

During the 32 seasonal epidemics, 285 882 ILI cases were reported among which 258 135 cases were described by SGPs (90.3%).

The average age of cases was 28.8 years (95% CI 28.7; 28.9) and the median was 26 years. Age of ILI cases was different according to dominant viruses (*P* value < 10^−10^). Compared to epidemics dominated by type B viruses (mean = 27.5 years; IC 95% 27.3; 27.7), ILI cases were on average older during epidemics dominated by A(H3N2) (mean = 30.6 years; IC 95% 30.3; 30.8), and younger for epidemics dominated by A(H1N1) (mean = 24.5 years; IC 95% 24.2; 24.8).

**Figure 4:**
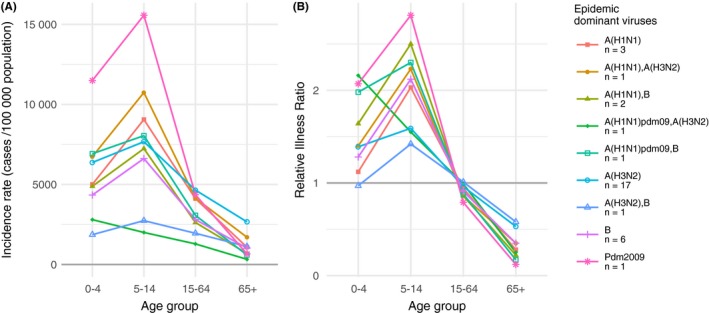
Age distribution of influenza epidemics grouped by viral dominant or co‐dominant (sub)types; (A) mean cumulative incidence rates; (B) mean relative illness ratio (RIR); from 1984/85 to 2016/17, *Sentinelles* network, France. Numbers of epidemics included for each series was reported in the legend

Overall, children of 5‐14 years had the highest attack rate with an average of 7352 cases per 100 000 population (95% CI 6187; 8516) regardless the dominant virus, followed by the <5 years (5545; 95% CI 4693; 6397), the 15‐64 years (3873, 95% CI 3269; 4477) and the elderly (1868; 95% CI 1392; 2344) (Figure 4A). Consistently across all dominant viruses, RIRs were highest in the young (<5 years and 5‐15 years) and decreased in adults with lowest values for the elderly (Figure 4B). School‐age children (5‐14 years) had higher RIR than youngest (<5 years) except for an epidemic co‐dominated by A(H1N1)pdm09 and B (2013/14) were RIRs were decreasing with age.

The sex ratio was consistent across seasonal epidemics, overall 51.0% (130 500/255 847) of the ILI patients being women.

Since the 1997/98 epidemic, on average, hospitalization was requested by the GP for 0.3% of ILI cases by epidemic (min = 0.1% in 2000/01, max = 0.6% in 2006/07), and for 2.1% of elderly ILI cases (≥65 years). There was no evidence of temporal trend from 1997/98 to 2016/17. However, hospitalization was more often requested for elderly during epidemics dominated by A(H3N2) (2.7%, IC 95% 2.3; 3.2 vs 1.2%, IC 95% 0.5; 1.9, *P* value < 10^−6^).

During the 2009 pandemic, mean age of cases was lower (21.1 years; 95% CI 20.8; 21.5) than all observed seasonal epidemics. Larger differences across age groups were also observed, RIR in young was higher than 2 (2.1 for <5 years (95% CI 1.1; 3.6); 2.8 for 5‐14 years (95% CI 2.0; 3.9)), while RIR in elderly was very low (0.1; 95% CI 0.0; 0.4) (Figure 4). The observed requested hospitalization was higher than for seasonal epidemics (1% of ILI cases).

## 4 DISCUSSION

Since 1984, the primary care *Sentinelles* surveillance network has monitored influenza epidemics in France. Its seniority, the stability of the methods and ILI definition used allowed the comparison of 32 seasonal influenza epidemics and a pandemic, observed between winters 1984/85 and 2016/17. We highlighted a decrease in epidemic size and height over time. We bring out associations between epidemics characteristics and circulating dominant or co‐dominant viruses: epidemic size, peak height and age of cases.

Influenza epidemic size and height observed in primary care decreased over time, especially among adults and elderly. The fourth largest epidemics were observed during the 80s, affecting more than 6% of the population by year (1984/85, 1985/86, 1988/89 and 1989/90). This decline was also observed in the Netherlands between 1986 and 2006,^26^ in the UK over 1966‐2006 27 and in New Zealand over 1997‐2006.^28^ We found that this decreased in size was not associated with a decrease in epidemic duration, which may suggest a reduction of transmissibility of influenza viruses. At first sight, this can be related to the influenza vaccination programme developed in France from the beginning of 80s, targeting first only elderly (≥75 years) and enlarged progressively to people aged 70 years and over and people with chronic conditions (in 1988 and then in 1991) and finally to people aged 65 years and over (in 2000).^29^ As a result, vaccine coverage of people aged 65 or over increased from 56% to 70% between 1989/90 and 2008/09 (Groupe d'Expertise et d'information sur la grippe, data not published), but this rate has declined after the 2009 pandemic.^30^ The French vaccination targets of 75% for all people at high risk 31, 32 had still not been achieved. Unlike to some others countries,^33^ influenza vaccination is not recommended for children in France, although we highlighted that the most affected age group was school‐age children (5‐14 years), regardless of the dominant viruses. For this age group, contact rates are the highest,^34^ contributing to a larger transmissibility and risk of infection.^35^ Although influenza vaccination might contribute to the declining trend in influenza epidemic size and height, its impact could be limited in France. Secondly, observed decrease in influenza incidence rates may also be related to behaviour changes in the population like changing lifestyles (reduced household size, less smoking, cleaner air) and hygiene improvements; which might have contributed to reduce the transmissibility of influenza viruses.^27^ Recently, the 2009 pandemic could have impacted hygiene habits as frequency of hand washing and use of alcohol‐based hand sanitizers which were strongly promoted during the epidemic period and probably remained more frequent since that time.^36^ Thirdly, changes in the biological properties of circulating influenza viruses may have occurred, particularly for A(H3N2) subtype 37—the most dominant virus during the seasons studied. The process of antigenic drifts might decrease affinity for human receptors and could affect the efficiency of infection and transmission.^37^ Lastly, changes related to consultations and reporting might have occurred over the 33‐year period. However, there were no modifications in the French health system that could have resulted in a significant shift of consulting patterns. In addition, since 1984, SGPs’ reports are electronically transmitted with the same rules and a similar form.

During the study period, type A viruses, and specially A(H3N2) subtype, were more often dominant in France, as it was already reported for Europe and USA.^38^ We highlighted that epidemics dominated by subtype A(H3N2) were mostly larger with older average age of cases and higher severity in elderly. This is consistent with greater virulence of type A,^39^ particularly for subtype A(H3N2), subject to more frequent changes in the major surface antigens, and responsible for epidemics with a greater morbidity and mortality especially among elderly.^40^, ^41^ This could be explained by acquired immunity against subtypes of influenza A(H1N1) in adults and elderly encountered through past exposure to epidemics,^40^, ^42^ while the A(H3N2) viruses evolve faster 43 where a lesser acquired immunity. Moreover, the greater severity of symptoms often associated with A(H3N2) infection 40 could increase adults and elderly's propensity to consult a GP. Type B epidemics were associated with lower epidemic size and occurred late, consistent with previous reports.^12^, ^44^ Only the 2012/13 epidemic dominated by B viruses was over 3500 cases per 100 000 population, where A(H1N1) and A(H3N2) were also circulating (25% and 20%, respectively).^12^

Our study has some limitations. We implemented a single influenza epidemic detection method among the various used worldwide.^45^ Based on its performances 20 and to ensure consistency with all data and scientific papers on the French *Sentinelles* network, we opted for the periodic regression used since 1991.^19^ A recruitment bias was possible as ILI cases reported by SGPs were not virologically confirmed and could be caused by other viruses than influenza. However, we used a very specific definition 11 and we included ILI cases only during the epidemic period—where influenza positivity rates of cases were the highest, allows to reduce this bias.^14^ Our study based on ILI consultations with a febrile ILI definition could underestimate true influenza burden in the community, especially among elderly who usually had lower symptoms (eg, low fever). Moreover, we did not include cases observed in nursing homes, where elderly could have worse health than those consulting in primary care. Conversely, influenza burden in children could be overestimated relatively to adults’ estimates as children may be more likely to consult a GP than adults.^36^ However, consistency of data collection since 1984,^10^ using the same ILI definition and epidemic detection method, allowed comparisons over time, as potential bias, if occurred, would be constant in time.

The main strength of our study is to rely on data collected by a long‐term surveillance system in primary care.^46^ For more than 30 years, ILI case definition remains unchanged as the electronic data collection protocol. This stability is needed to compare influenza activity and epidemiology over several decades in the community: characterization of epidemics, description of cases, understanding of past patterns, monitoring change in trends and estimating influenza vaccine effectiveness.^14^, ^18^ Long‐standing surveillance systems constitute an important and reliable source of information for epidemiological research, to plan for the pressure on health services and to adapt and develop influenza control strategies in the coming years.

In conclusion, data collected by the French *Sentinelles* network since 1984 allowed us to highlight a declining trend in influenza epidemic size and height. This decrease could be related to several factors including increased vaccine coverage, hygiene improvements and diminished fitness of influenza viruses. Future researches combining epidemiological and virological issues should help to identify and quantify impact of contributing factors to this observed decrease.

## CONFLICT OF INTEREST

None declared.
